# Neferine alleviates chronic stress-induced depression by regulating monoamine neurotransmitter secretion and gut microbiota structure

**DOI:** 10.3389/fphar.2022.974949

**Published:** 2022-09-02

**Authors:** Zaiquan Dong, Qinglian Xie, Feiyu Xu, Xiaoling Shen, Yanni Hao, Jin Li, Haizhen Xu, Qiang Peng, Weihong Kuang

**Affiliations:** ^1^ Mental Health Center of West China Hospital, Sichuan University, Chengdu, China; ^2^ Department of Psychiatry and National Clinical Research Center for Geriatrics, West China Hospital, Sichuan University, Chengdu, China; ^3^ Department of Outpatient, West China Hospital, Sichuan University, Chengdu, China; ^4^ West China School of Medicine, Sichuan University, Chengdu, China; ^5^ State Key Laboratory of Oral Diseases, National Clinical Research Center for Oral Diseases, West China Hospital of Stomatology, Sichuan University, Chengdu, China

**Keywords:** neferine, anti-depressant, chronic mild stress, intestinal flora, Lactobacillus, monoamine neurotransmitters

## Abstract

Neferine (Nef) might possess anti-depressive properties; however, its therapeutic effects are yet to be elucidated. Therefore, in this study, we aimed to explore the anti-depressant property of Nef using a mouse model of chronic stress-induced depression. Fifteen depression-prone mice were randomly selected and divided into three groups, namely, the model, Nef, and fluoxetine (Flu) groups. We observed that in tail suspension and forced swimming tests, the Nef and Flu treatments significantly decreased the immobility time of the depressed mice, and increased their sucrose preference indices. Moreover, both Nef and Flu treatments induced significant increases in the levels of anti-depressant neurotransmitters, including dopamine (DA), serotonin (5-HT), and norepinephrine (NE), and also reduced pathological damage to the hippocampus of the depressed mice. Incidentally, Illumina MiSeq sequencing analysis demonstrated that the relative abundance of *Lactobacillus* in the intestinal microbiota of depressed mice was restored after Nef/Flu treatment. Moreover, colonic *Lactobacillus* abundance was positively correlated with the levels of DA, 5-HT, and NE in the hippocampus of the mice. In conclusion, Nef improved monoamine neurotransmitter secretion and modulated the intestinal flora structure, particularly the abundance of *Lactobacillus*. Hence, it showed considerable anti-depressant potential, and might be a prospective anti-depressant therapeutic agent.

## Introduction

In recent years, the incidence of depression, especially among children and adolescents, has been on the rise. In fact, depression has become the second most common cause of death among young (Patton et al., 2009; Johnson et al., 2018; Christ et al., 2020; Arzola et al., 2022). Therefore, conducting research to determine effective treatment options for depression is of great social significance.

Based on the “monoamine hypothesis,” a decrease in monoamine neurotransmitter levels in the synaptic cleft is recognized as the major factor related to the pathogenesis of depression in clinical settings (Zhang et al., 2019). Most classical antidepressants, such as tricyclic antidepressants (TCAS), tetracyclic antidepressants (HCA), monoamine oxidase inhibitors (MAOI), and selective serotonin (5-HT) reuptake inhibitors (SSRIs), which are all commercially available, are based on this theory (Zhang et al., 2019). However, despite using adequate doses of these drugs as well as maintenance therapy, 30%–40% of patients remain unresponsive, leading to significant treatment resistance and unsatisfactory outcomes (Cipriani et al., 2018).

In both human and animal experimental models, stress has been widely recognized as an independent risk factor for the occurrence of major depressive disorder (MDD) (Yirmiya et al., 2015; Cruz-Pereira et al., 2020). Additionally, stress-related factors, such as infection, inflammation, hypoxia, or psychological stress can lead to depression *via* the activation of the hypothalamic-pituitary-adrenal axis or the autonomic nervous system, the inhibition of afferent vagus nerve fibers, and leading to consequently, inflammation and tryptophan metabolism dysregulation (Herselman et al., 2022). Stress may also lead to functional deficits in certain brain regions, three of which, including the prefrontal cortex, hippocampus, and amygdala, have received the most attention in depression-related studies (Arzola et al., 2022). The notable hallmarks of MDD include apparent structural and functional deficits in the hippocampus, a brain region that is linked to mood regulation and memory (Yun et al., 2018). Depressed humans and chronically-stressed animals have reduced hippocampal activity and volumes, and show decreased expression of activity-dependent genes and processes, including reduced adult neurogenesis in the hippocampus (Kronmüller et al., 2008; Rosa and Lisanby, 2012; Chevalier et al., 2020; Liu et al., 2021). Therefore, these hippocampal deficits have long been the target of depression treatments in an effort to ameliorate depression symptoms (Miller and Hen, 2015; Yun et al., 2016; Chevalier et al., 2020; Liu et al., 2021).

Gut microbiota play an important role in regulating host brain development and behavior (Mcguinness et al., 2022; Tian et al., 2022). Several studies have demonstrated that gut microbiota dysbiosis is closely related to host’s depression symptoms (Jiang et al., 2015; Petra et al., 2015; Feng et al., 2022; Ortega et al., 2022). Interestingly, a previous study revealed that regulating the composition of intestinal flora might be effective for preventing as well as treating depression (Yarandi et al., 2016). This may be because intestinal flora can influence the absorption and bioavailability of oral drugs (Klünemann et al., 2021; Shang et al., 2021). Other recent studies have suggested that stress can also contribute to MDD by affecting gut microbiota (Herselman et al., 2022). This is because stress can lead to impaired gut barrier integrity and reduced gut mucus, as well as gut microbial dysbiosis, which in turn increases neuroinflammation-induced depression- and anxiety-like behaviors (Guolan et al., 2018; Amini-Khoei et al., 2019; Oh et al., 2020; Deng et al., 2021; Gao et al., 2022). Therefore, improving gut dysbiosis may become a new strategy for the treatment of stress-induced MMD.

Recently, attempts have been made at using traditional Chinese medicine to treat MDD by altering gut microbiota structure (Li et al., 2022; Song et al., 2022; Tan et al., 2022). For example, the antidepressant, Shuganjieyu Capsules can alter gut microbiota structure and function in stress-induced depressed rats, and improve depressive symptoms (Tan et al., 2022). Interestingly, in addition to its use as treatment for nervous disorders, high fever, agitation, and insomnia, the seed embryo of lotus, a common traditional Chinese medicine, has anti-hypertensive and sedative properties (Kumarihamy et al., 2015). Specifically, neferine (Nef), derived from the seed embryo of lotus, is a unique bisbenzylisoquinoline alkaloid (Marthandam Asokan et al., 2018). A previous study on diabetic mice revealed that Nef exerts neuroprotective effects as well as ability to improve memory and overcome cognitive impairment (Wu et al., 2020). It has also been reported that Nef can be used to reverse cognitive impairment in Alzheimer’s disease in rats (Yin et al., 2020). Additionally, Nef has affinity for δ- and μ-opioid receptors (Kumarihamy et al., 2015). Moreover, it has also been demonstrated that opioid peptides and their receptors are potential candidates for developing novel antidepressant therapies; their effects are mediated by three receptor subtypes, namely δ-, μ-, and κ-receptors (Berrocoso et al., 2009). Previous studies have also shown that Nef has sedative and anxiolytic effects and can significantly reduce locomotor activity in mice in the forced swimming test (Sugimoto et al., 2008; Sugimoto et al., 2010); thus, it has been speculated that it may have antidepressant effects. Studies have shown that Nef shows anti-inflammatory activity (Jung et al., 2010; Deng et al., 2021), and inflammatory response is an important mechanism for the pathogenesis of depression (Liu et al., 2017). Other studies have demonstrated that it exerts antioxidant stress effects to reduce inflammatory response (Bharathi Priya et al., 2021), and the regulatory pathways in this regard include the ROS/NLRP3/Caspase-1 (Takahashi et al., 2019; Tang et al., 2019), NF-κB (Zhong et al., 2020; Chiu et al., 2021), and PI3K/AKT/mTOR (Qi et al., 2021) signaling pathways. Further, given that several studies on the use of Nef to regulate oxidative stress and inflammatory response in other models have been reported, in this study, our desire was to investigate the therapeutic effect of Nef on depression from another perspective, such as the monoamine hypothesis and intestinal flora structure.

Therefore, our aim in this study was to explore the anti-depressive effects of Nef *via* behavioral analyses, hippocampal neurotransmitter level assessment, and the investigation of the structural changes in the intestinal flora of mice. We also performed a preliminary exploration of the intestinal flora targets for the anti-depressive functions of Nef. Thus, we demonstrated the regulatory mechanism of the “microbiota-gut-brain” axis in depression.

## Materials and methods

### Experimental animals and study groups

We purchased 30 C57BL/6J mice (6-week-old, male) from Chengdu Dasuo Biotechnology Co., Ltd. (Sichuan, China), of which 25 were randomly selected for the establishment of the depression model, while the remaining five served as the control group (control). All the mice were housed and maintained under a 12-h/12-h light-dark cycle at 20–24°C. The depression model was established using a protocol in which the mice were raised alone and exposed to chronic unknown mild stress (CUMS) daily for 8 weeks, as described in a previous study (Kim et al., 2020). The specific methods are shown in Tables 1, 2. After 8 weeks, the mice were then subjected to behavioral tests, and the ones with significant depressive symptoms were considered successful depression models. Subsequently, 15 depression-prone mice were randomly selected and divided into three groups, namely the model, Nef, and fluoxetine (Flu) groups (*n* = 5 per group). The mice in the Nef group were intraperitoneally administered Nef injections at 20 mg/kg/d for 4 weeks, while those in the Flu group were intraperitoneally administered Flu injections at 20 mg/kg/d for 4 weeks. Nef (CAS. 2292-16-2, purity ≥98%) was purchased from Sichuan Weikeqi Biological Technology, Co., Ltd. (Sichuan, China), while Flu was purchased from Sigma Aldrich (St. Louis, MO, United States). Moreover, the mice in the control and model groups were intraperitoneally administered equal amounts of normal saline for 4 weeks. All the procedures involving the animals were approved by the ethics committee of West China Hospital (WCH) of Sichuan University (approval number 20211707A).

**TABLE 1:** Chronic unknown mild stress (CUMS).

Number	Treatments (duration)
1	Continuous overnight illumination (12 h)
2	Intermittent illumination (light on and off every 1 h; 3 h)
3	Paired cage (2–3 animals in each cage; 3 h)
4	Empty cage housing (18 h)
5	Physical restraint (2 h)
6	45° cage tilt (3 h)
7	Water deprivation (24 h)
8	Food deprivation (24 h)
9	Tail nip (1 min)
10	White noise overnight (80–85 dB; 3 h)
11	Wet bedding (400 ml water in 200 g sawdust bedding; 18 h)

**TABLE 2:** Chronic unknown mild stress (CUMS) schedule.

Cycle	Treatments										
Cycle 1	1	2	3	4	5	6	7	8	9	10	11
Cycle 2	3	1	10	5	11	8	4	7	2	6	9
Cycle 3	2	10	11	5	4	8	7	6	9	1	3
Cycle 4	8	4	3	6	10	1	11	2	7	5	9
Cycle 5	11	6	4	3							

### Behavior test

#### Sucrose preference test (SPT)

A day before sampling, SPTs were performed. As previously described (Liu et al., 2020), the SPTs were performed in two phases, i.e., the adaptation training phase, followed by the test phase. During the first phase, all the mice were trained to become adapted to drinking sucrose water. Thereafter, the mice were deprived of this water as well as food for 24 h. Then in the test phase, the mice were allowed to choose between two bottles, one containing 1% (W/V) sucrose solution and the other containing pure water. Both bottles were weighed in advance. After 12 h, both bottles were removed from the experimental set-up and reweighed. The total liquid, sucrose solution, and pure water consumptions of the mice were then recorded. The SPT results thus obtained were then used to measure anhedonic responses.

The formula for calculating sugar preference (SP) index was as follows:

Sp index(%)=[Sucrose solution consumption/(Sucrose solution consumption+Pure water consumption)]×100 (1)

#### Tail suspension test (TST)

On the second day after the SPT, the TST, a behavioral despair-based test, was performed. As previously described (Steru et al., 1985; Rosa et al., 2019), the mice were suspended 15 cm above the floor using an adhesive tape, which was placed approximately 1 cm from the tip of the tail. Thereafter, the duration of immobility of the mice in the 4 min test was recorded.

#### Forced swimming test (FST)

Three hours after the TST, the FST, another behavior despair-based test, was performed. As previously described (Liu et al., 2020), the mice were placed in a cylindrical container with clean water. The water depth was 30 cm, and the temperature was 25 ± 1°C. The mice were forced to swim, and the durations of their immobile states within the last 4 min of an 8 min test period were recorded.

### Sample collection

After behavior assessment, the mice were intraperitoneally anesthetized with 30 mg/kg sodium pentobarbital and sacrificed. Hippocampal tissue samples and colon contents were then collected for subsequent analyses. A portion of the hippocampal tissue was fixed in 4% paraformaldehyde for pathological examination, while the rest was preserved in liquid nitrogen for enzyme-linked immunosorbent assay (ELISA). The colon contents were stored in liquid nitrogen and sent to Shanghai Personal Biotechnology Co., Ltd. (Shanghai, China) for 16S rRNA high-throughput sequencing.

### Hematoxylin and eosin (H&E) staining

Hippocampal tissue samples from the mice were subjected to H&E staining according to the standard procedure. In brief, the hippocampal tissue samples were fixed with 4% paraformaldehyde, embedded in paraffin blocks, dehydrated, and sliced. Subsequently, the sections were stained with hematoxylin for 10–20 min, followed by eosin for 3–5 min. The staining results were then observed (at ×100 and ×400 magnification) using the Motic BA210 digital tri camera microscope (Xiamen, China).

### Nissl staining

Hippocampal tissue samples were also analyzed *via* Nissl staining according to the standard procedure. In brief, the hippocampal tissue sections were placed in 1% toluidine blue solution (50°C) at 56°C for 20 min. After differentiating and dehydrating in different concentrations of alcohol, staining was then observed (at ×400 magnification) using the Motic BA210 digital tri camera microscope (Xiamen, China). The average optical density of five sections for each region was recorded as the final value for that region.

### ELISA

The levels of hippocampal dopamine (DA), serotonin (5-HT), and norepinephrine (NE) were determined using ELISA kits according to the manufacturer’s instructions. These ELISA kits for DA (ml002024), 5-HT (ml001891), and NE (ml063805) were purchased from Shanghai Enzyme-linked Biotechnology Co., Ltd. (Shanghai, China).

### 16S rRNA Illumina MiSeq sequencing

The colonic contents, which were stored in liquid nitrogen and sent to the Shanghai Personal Biotechnology Co. Ltd., were subjected to paired-end sequencing using the 16S rRNA Illumina MiSeq platform for gene sequencing (Illumina, San Diego, CA, United States). After denoising, QIIME2 (2019.4) software was used for taxonomic annotation. The “Qiime taxa Barplot” command was launched, and the feature table was generated, after singleton data removal, to visualize the distribution of the microbiota composition for each sample at both the phylum and genus levels. Further, the analysis results were presented using histograms.

### Statistical analyses

Data were analyzed using GraphPAD software version 8 (GraphPad Software Inc., San Diego, CA, United States), and the results were expressed as the mean ± standard deviation (SD). Statistical differences among the control, model, Nef, and Flu groups were determined by performing one-way analysis of variance (ANOVA). If the data fitted the homogeneity of variance, then the least significant difference (LSD) analysis was performed; otherwise, Tamhane’s T2 analysis was performed. Additionally, Pearson correlation analysis were performed using SPSS software version 19.0 (IBM, Armonk, NY, United States). Statistical significance was set at *p* < 0.05.

## Results

### Effects of Nef on depressed mice behavior

**FIGURE 1:**
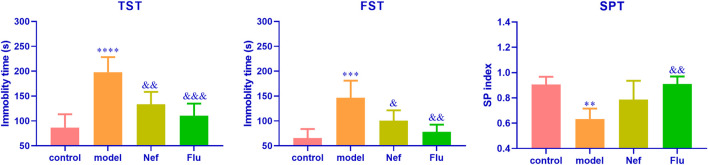
Nef improves behavioral depression symptoms in mice models of depression. TST, tail suspension test; FST, forced swimming test; SPT, sucrose preference test; Nef, neferine; Flu, fluoxetine. Nef and Flu both improved the depressed behavior of depressed mice. Data are presented as mean ± standard deviation (SD; *n* = 5 per group). ^**^ *p* < 0.01, ^***^ *p* < 0.001, ^****^ *p* < 0.0001 vs control; ^&^ *p* < 0.05, ^&&^ *p* < 0.01, ^&&&^ *p* < 0.001 vs model.

As shown in Figure 1, in both the TST (*p* < 0.0001) and FST (*p* < 0.001), the model mice showed significantly increased immobility durations, while their SP indices decreased significantly (*p* < 0.01) relative to those of the control mice. These observations indicated the successful establishment of the mouse model of depression. Additionally, the immobility durations of the Nef- and Flu-treated depressed mice in the TST (Nef, *p* < 0.01; Flu, *p* < 0.001) and FST (Nef, *p* < 0.05; Flu, *p* < 0.01) were significantly decreased, while their SP indices were significantly increased (Nef, *p* > 0.05; Flu, *p* < 0.01) relative to those of the model mice.

### Effects of Nef on depression-related factors in the hippocampus of depressed mice

**FIGURE 2:**
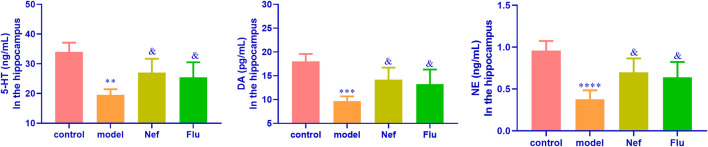
Hippocampal expression of anti-depression factors in depressed mice restored by Nef treatment. DA, dopamine; 5-HT, serotonin; NE, norepinephrine; Nef, neferine; Flu, fluoxetine. Nef and Flu both increased the levels of DA, 5-HT, and NE in the hippocampus of depressed mice. Data are presented as mean ± standard deviation (SD; *n* = 5 per group). ^**^ *p* < 0.01, ^***^ *p* < 0.001, ^****^ *p* < 0.0001 vs control; ^&^ *p* < 0.05 vs model.

As shown in Figure 2, the hippocampal levels of anti-depression factors, namely DA (*p* < 0.01), 5-HT (*p* < 0.001), and NE (*p* < 0.0001), were significantly reduced in the model mice compared with their levels in the hippocampus of the control mice. Incidentally, the Nef and Flu treatments led to a significant increase in the hippocampal levels of DA (Nef, *p* < 0.05; Flu, *p* < 0.05), 5-HT (Nef, *p* < 0.05; Flu, *p* < 0.05), and NE (Nef, *p* < 0.05; Flu, *p* < 0.05) in the depressed mice, compared with their corresponding levels in the model mice.

### Effects of Nef on pathological damage of hippocampal tissue in depressed mice

**FIGURE 3:**
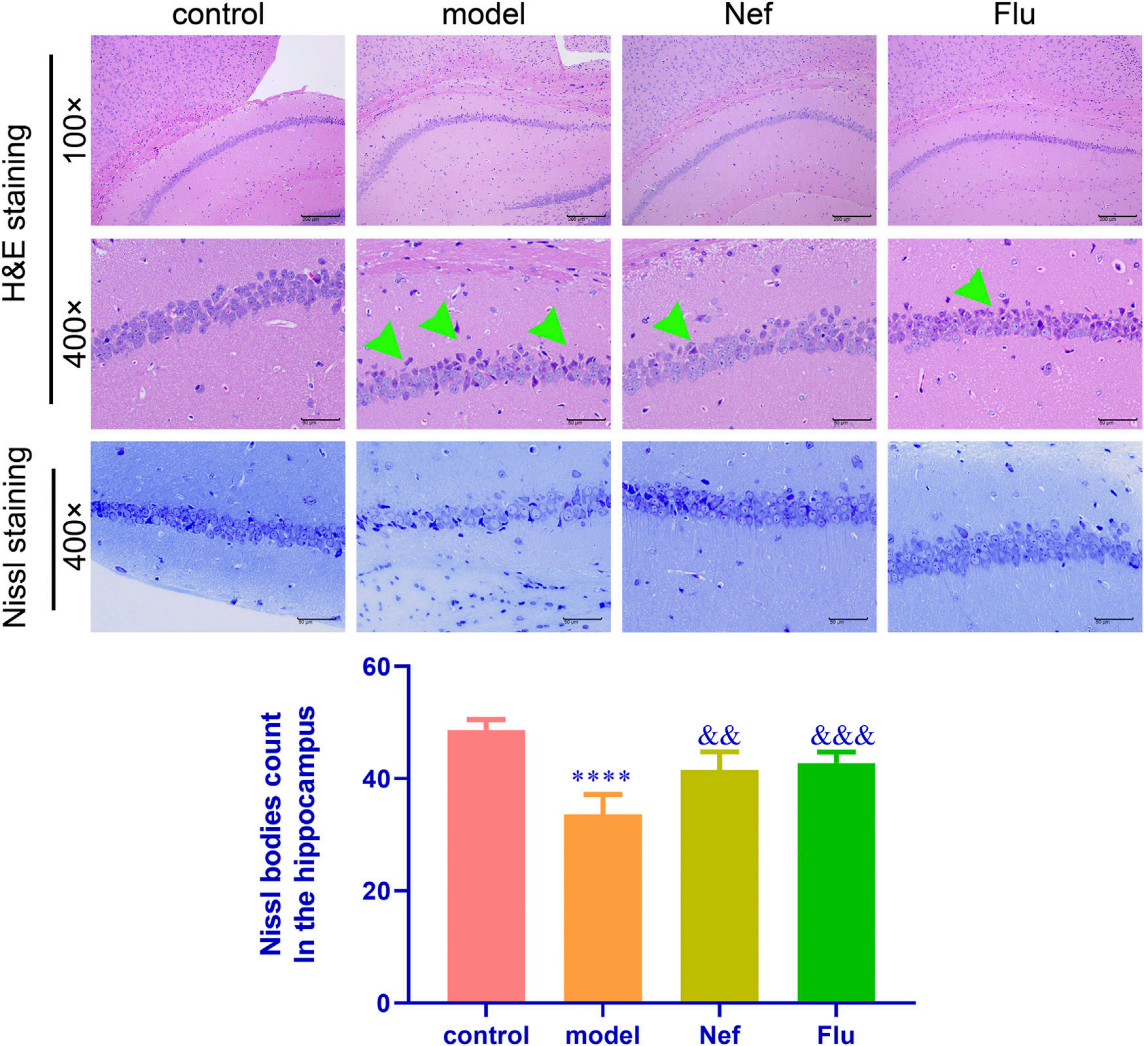
Nef alleviates pathological damage in the hippocampus of depressed mice. H&E, hematoxylin and eosin; Green arrow, necrotic pyramidal cells; Nef, neferine; Flu, fluoxetine. Both Nef and Flu both reduced pyramidal cell necrosis and increased Nissl body count in the hippocampus of depressed mice. Data are presented as mean ± standard deviation (SD; *n* = 5 per group). ^****^ *p* < 0.0001 vs control; ^&&^ *p* < 0.01, ^&&&^ *p* < 0.001 vs model.

As shown in Figure 3, relative to the control mice, the hippocampus of depressed mice showed significantly increased pyramidal cell necrosis, while the number of Nissl bodies (*p* < 0.0001) decreased significantly. However, both the Nef and Flu treatments significantly reduced pyramidal cell necrosis and increased the number of Nissl bodies in the depressed mice (Nef, *p* < 0.01; Flu, *p* < 0.001) relative to the model mice.

### Effects of Nef on gut microbiota of depressed mice

**FIGURE 4:**
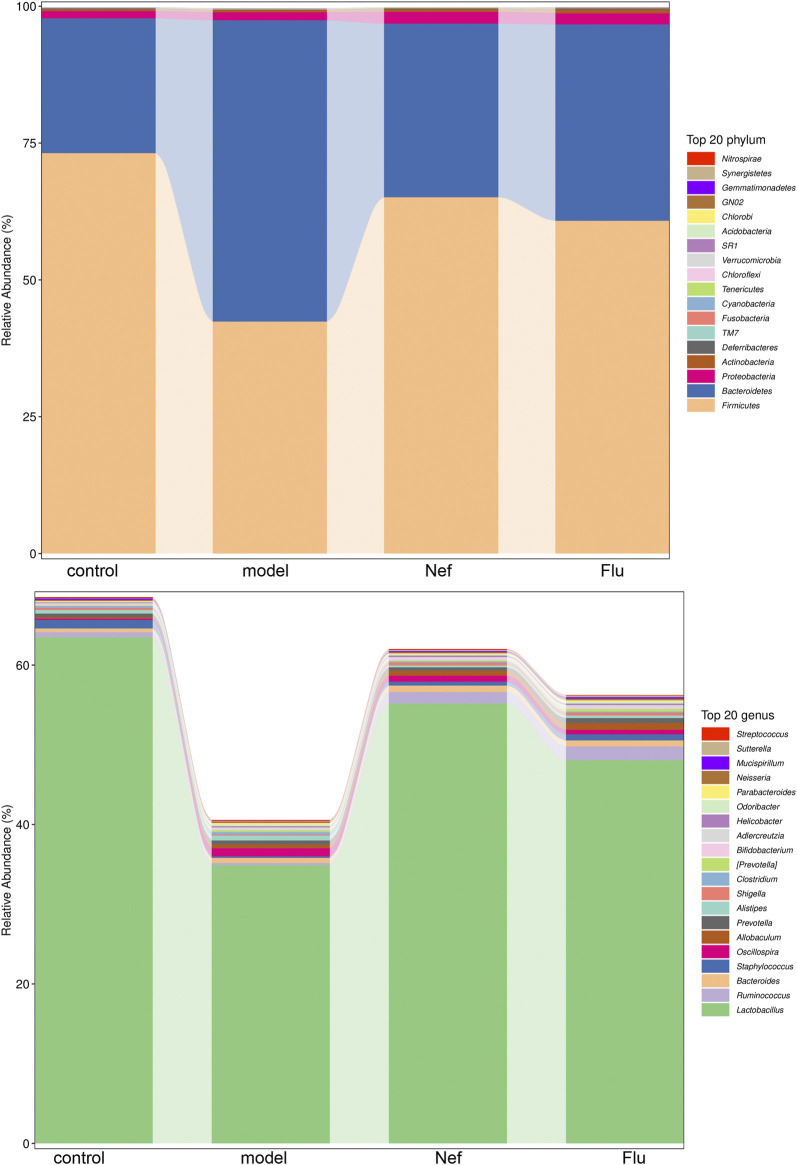
Nef treatment improves gut microbiota in depressed mice. The figure depicts gut microbiota composition in mice at phylum and genus levels (*n* = 5 per group). Nef, neferine; Flu, fluoxetine. At the phylum level, both Nef and Flu treatments reverted the changes in the relative abundances of *Firmicutes* and *Bacteroidetes* in the depressed mice. At the genus level, Nef as well as Flu treatments also restored the relative abundance of *Lactobacillus* in depressed mice.

The results of Illumina MiSeq sequencing of the colonic content of the mice are presented in Figure 4. At the phylum level, compared with the control group, the model group showed a decrease in the relative abundance of *Firmicutes* and an increase in that of *Bacteroidetes*. Furthermore, both Nef and Flu treatments reverted these changes in the relative abundances of *Firmicutes* and *Bacteroidetes* in the depressed mice. Additionally, at the genus level, *Lactobacillus* was the most dominant microflora in all the groups; however, compared with the control group, the model group showed a decrease in the relative abundance of this genus. Incidentally, both the Nef and Flu treatments restored the relative abundance of *Lactobacillus* in the depressed mice.

**FIGURE 5:**
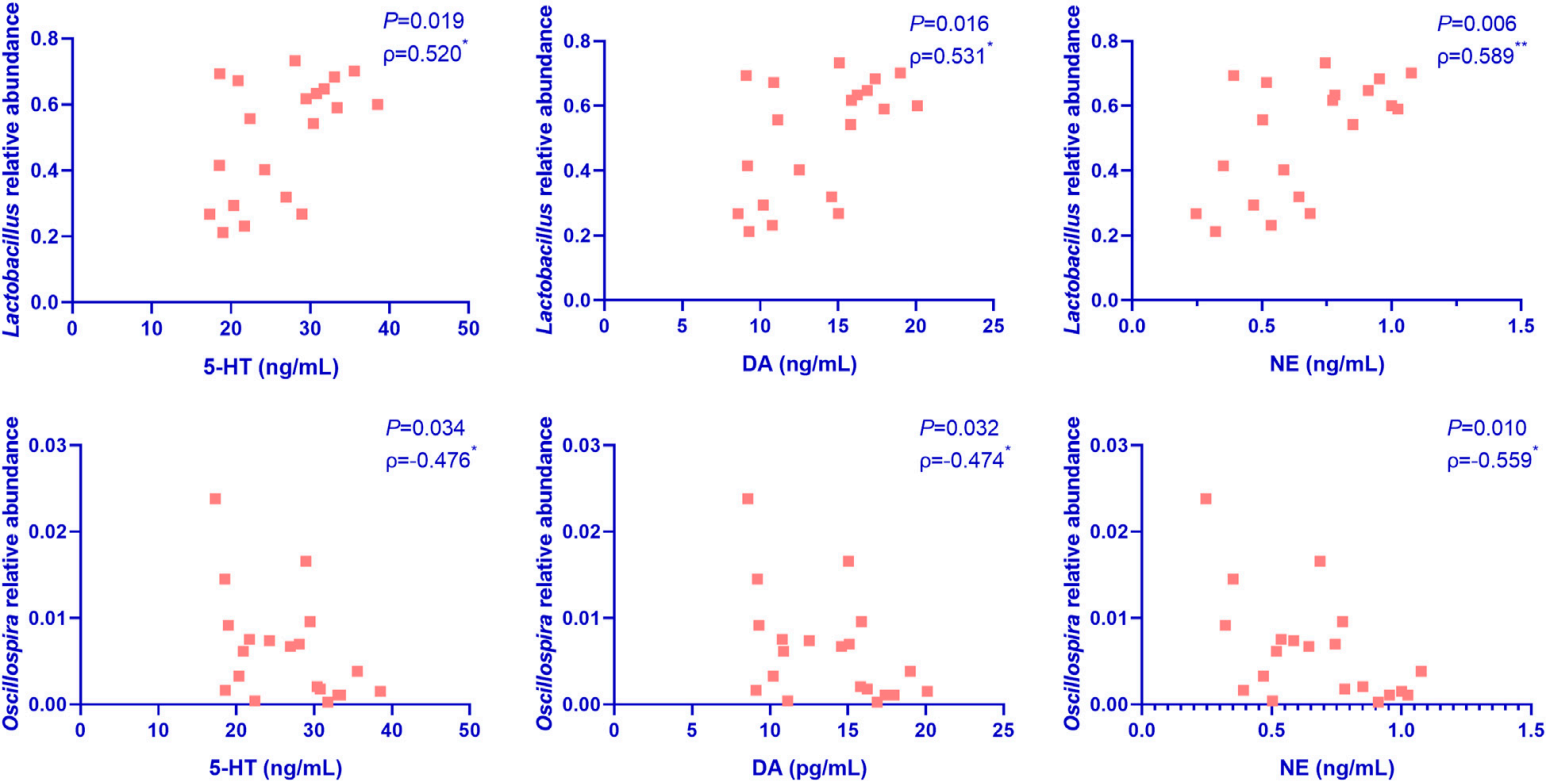
Pearson correlation analysis of the correlation between the top 20 dominant microflora at the genus level and the hippocampal levels of anti-depressant factors in mice (*n* = 5 per group). Nef, neferine; Flu, fluoxetine; DA, dopamine; 5-HT, serotonin; NE, norepinephrine. The relative abundance of *Lactobacillus* was significantly positively correlated with the hippocampal levels of DA, 5-HT, and NE, while that of *Oscillospira* showed significantly negatively correlations in this regard.

To further confirm the correlation between gut microbiota and the improvement of depression symptoms, we performed Pearson correlation analysis involving the top 20 dominant microflora at the genus level and the hippocampal levels of anti-depressant factors. As shown in Figure 5, the relative abundance of *Lactobacillus* was significantly positively correlated with hippocampal levels of the anti-depressant factors, DA, 5-HT, and NE, while that of *Oscillospira* showed significantly negative correlations in this regard.

## Discussion

In view of the current situation that existing treatments for MDD still lack satisfactory efficacy, it is of great practical significance to continuously explore new and better treatment options. In this regard, studies on the antidepressant effects of traditional Chinese medicine represent one of the possible research directions in this regard.

In this study, Nef treatment reduced the immobility durations of depressed mice in TST and FST and increased their SP indices, suggesting that Nef played an anti-depressant role in a state of depression. In fact, Nef has been studied for its anxiolytic and antidepressant effects. In an elevated plus maze test, Sugimoto et al. (2008) observed that Nef exerts anxiolytic effects in mice. Based on FST results, they also indicated that Nef exhibits antidepressant-like antidepressant effects in mice, similar to typical antidepressants, and these effects were found to be mediated by 5-HT1A receptors (Sugimoto et al., 2010; Shi et al., 2019). Similar to these previous studies, our findings reconfirmed the antidepressant effect of Nef.

Our results also revealed that Nef improved depression by reducing hippocampal pyramidal cell necrosis and alleviating hippocampal lesions. Reportedly, pyramidal cell damage is associated with depression and memory decline (Salim, 2017). Several treatment approaches like Tuina, which affect the activation and functional connectivity of the hippocampus are thought to improve depressive symptoms (Tao et al., 2022). Therefore, the reason why Nef can protect hippocampal cells against lesions may be that it exerts anti-oxidative stress effects or inhibits the excessive release of the neurotransmitter glutamate, thereby exerts a protective effect on nerve cells (Marthandam Asokan et al., 2018; Yeh et al., 2020). However, given that related studies are limited, the specific mechanism by which Nef exerts its protective effect on nerve cells needs to be further studied.

The results of this study also demonstrated that Nef treatment restored the hippocampal levels of 5-HT, NE, and DA, thereby indicating that Nef exerts an anti-depressive effect *via* 5-HT/NE/DA triple reuptake. Given the critical roles of 5-HT, NE, and DA in the pathogenesis of MDD (Maletic et al., 2007; Liu et al., 2020; Rominger et al., 2015; Zhong et al., 2020), it is reasonable to think that this should be an important mechanism for the antidepressant efficacy of Nef. Zhao et al. (2022) also reported that another herbal medicine, Xiebai glycosides, can significantly improve the levels of NE and DA in brain homogenate from depressive model rats. Studies have also confirmed that the antidepressant effect of Nef is mediated by the 5-HT1A receptor. Specifically, Nef may enhance the activity of 5-HT neurons by inhibiting 5-HT reuptake or activating 5-HT metabolism (Sugimoto et al., 2010; Sugimoto et al., 2015). However, the mechanisms by which it affects DA and NE function are still unclear, hence require further in-depth studies.

In recent years, a large number of studies have confirmed that the gut microbiota composition and the associated metabolites are related to the pathogenesis, clinical phenotype, and treatment effect of depression (Mayneris-Perxachs et al., 2022), and even the structure of the brain (Lee et al., 2022). Colonization by gut microbiota from patients with depression can lead to depression-like behaviors in mice (Kelly et al., 2016; Zheng et al., 2016). However, probiotics, exercise, and diet can affect gut microbiota structure and also show antidepressant potential (Donoso et al., 2022). Additionally, both animal and human studies have shown that alterations in gut microbial composition and metabolic function may be associated with differential responses to antidepressants in depression (Duan et al., 2021; Zhang et al., 2021; Dong et al., 2022). In a particular study, it was observed that polyphenols in an edible herbal medicine can alter the abundance of flora associated with neuroinflammation by reversing intestinal microbiota dysbiosis and that intestinal flora-mediated chemical modification of polyphenols can result in their conversion into active secondary metabolites that improve depression (Hao et al., 2022). Another study showed that resveratrol markedly increases brain derived neurotrophic factor (BDNF) expression in the hippocampus, and this can help to improve depression and anxiety symptoms (Yu et al., 2019). Our study demonstrated that both Nef and Flu treatments increased the relative abundances of species belonging to phylum *Firmicutes*, but decreased those of species belonging to phylum *Bacteroidetes* in the depression mouse model. Additionally, they increased the relative abundance of *Lactobacillus* at the genus level. These results suggested that Nef and Flu treatments might improve depression *via* intestinal flora. Further, Pearson correlation analysis indicated that the relative abundance of *Lactobacillus* was significantly positively correlated with the levels of DA, 5-HT, and NE in the hippocampus, while that of *Oscillospira* showed significantly negatively correlations in this regard, suggesting that Nef improved depression *via* the brain-gut-microbial axis. Hence, *Lactobacillus* and *Oscillospira* might be the key microbial species associated with anti-depression.

Reportedly, *Lactobacillus* can mediate anti-depressant effects by promoting the functions of various neurotransmitters, such as 5-HT, DA, NE, and gamma-aminobutyric acid (GABA) (Yong et al., 2019; Yunes et al., 2020). The hypothalamic–pituitary–adrenal (HPA) axis is another target of *Lactobacillus* for treating depression symptoms (Johnson et al., 2021). Specifically, *Lactobacillus rhamnosus* can alter the expression of central GABA receptors, increase GABA expression level, and downregulate the HPA axis through the vagus nerve pathway, thereby functioning as an anti-depressant (Bravo et al., 2011; Janik et al., 2016). Therefore, the anti-depressant effects of Nef and Flu might be mediated through *Lactobacillus*, which is part of the intestinal flora of depressed mice; however, this requires further investigation.

In conclusion, this study revealed that Nef exerts therapeutic effects on depressed mice owing to its ability to improve hippocampal nerve damage, alleviate anti-depressant neurotransmitter secretion, and enrich the gut microbiota structure. Specifically, *Lactobacillus* might be the gut microbial target of Nef in treating the symptoms of depression.

## Data Availability

The datasets presented in this study can be found in online repositories. The names of the repository/repositories and accession number(s) can be found below: https://www.ncbi.nlm.nih.gov/bioproject/PRJNA857835.
